# Insight into the biochemical and physiological mechanisms of nanoparticles-induced arsenic tolerance in bamboo

**DOI:** 10.3389/fpls.2023.1121886

**Published:** 2023-03-31

**Authors:** Abolghassem Emamverdian, Yulong Ding, Mirza Hasanuzzaman, James Barker, Guohua Liu, Yang Li, Farzad Mokhberdoran

**Affiliations:** ^1^ Co-Innovation Center for Sustainable Forestry in Southern China, Nanjing Forestry University, Nanjing, China; ^2^ Bamboo Research Institute, Nanjing Forestry University, Nanjing, China; ^3^ Department of Agronomy, Faculty of Agriculture, Sher-e-Bangla Agricultural University, Dhaka, Bangladesh; ^4^ School of Life Sciences, Pharmacy and Chemistry, Kingston University, Kingston-upon-Thames, United Kingdom; ^5^ Department of Mathematical Sciences, Florida Atlantic University, Boca Raton, FL, United States

**Keywords:** arsenic, bamboo plant, zinc oxide nanoparticle, silicon dioxide nanoparticle, titanium dioxide nanoparticle, tolerance index

## Abstract

**Introduction:**

Arsenic (As) contamination in soil, sediments, and water poses a significant threat to the growth of bamboo plants. However, nanoparticles with high metal absorbance capacity can play a key role in the reduction of heavy metals toxicity in plants as well as maintaining their growth under toxicity.

**Methods:**

Hence, an *in vitro* experiment was conducted to determine the influence of three types of nanoparticles: 150 µM silicon nanoparticles (SiO_2_ NPs), 150 µM titanium nanoparticles (TiO_2_ NPs), and 150 µM zinc oxide nanoparticles (ZnO NPs) on As (150 µM and 250 µM) tolerance enhancement of a one-year-old bamboo species (*Pleioblastus pygmaeus*).

**Results and discussion:**

The results showed that while As at 150 µM and 250 µM significantly disrupted the plant growth by excessive generation of reactive oxygen species (ROS) components, and inducing cell membrane peroxidation, the addition of NPs increased both enzymatic and non-enzymatic antioxidant activities, upregulated glyoxalase defense system, and improved gas exchange parameters and photosynthetic pigments content, leading to the enhanced plant shoot and root dry weight. These were achieved by lowering levels of ROS, electrolyte leakage (EL), malondialdehyde (MDA), hydrogen peroxide (H_2_O_2_) and the superoxide radical ( ${\text{O}}_{2}^{•-}$ ), as well as decreasing As accumulation in the plant organs. Thus, it might be concluded that ZnO NPs, SiO_2_NPs, and TiO_2_NPS alone or in combination can significantly increase the bamboo plant tolerance to As toxicity *via* key mechanisms, including induction of various antioxidants and glyoxalase defense systems, scavenging of ROS and methylglyoxal (MG), increasing phytochelatins production, reduction of As accumulation and translocation, and improving photosynthetic pigments under As toxicity. Additionally, the results showed that the combined application of 150 µM ZnO NPs, SiO_2_ NPs, and TiO_2_ NPs had the greatest effect on enhancing the plant tolerance to As at 150 µM and 250 µM.

## 1 Introduction

Arsenic (As) is naturally present in the environment, however, its bioavailability has been increasing by different anthropogenic-manufactured compounds, such as herbicides, insecticides, and pesticides, leading to a rise in As contamination levels in groundwater and agricultural soils. (Garg and Singla, 2011; Hughes et al., 2011). According to a report by the World Health Organization (WHO), approximately 200 million people worldwide are exposed to As toxicity. The maximum concentration of As in drinking water is reported to be ≈10 μg L^−1^ (Khalid et al., 2017; Abbas et al., 2018). Due to its great mobility, As can quickly affect plants and crops. In addition to its risks to human health, As can hinder plant growth and development (Garg and Singla, 2011). It is reported that the overproduction of ROS by As induces oxidative stress, which occurs during the conversion of As (V) to As (III), resulting in increased concentration of free radicals and non-radicals in plant cells, such as superoxide radicals ( ${\text{O}}_{2}^{•-}$ ), hydrogen peroxide (H_2_O_2_), and hydroxyl radicals (Sharma et al., 2012; Armendariz et al., 2016; Shahid et al., 2017). As (III) has greater mobility, solubility, and toxicity relative to As (V) (Caporale and Violante, 2016; Ji et al., 2017). Arsenic has detrimental effects on the morphology, physiology, and growth of plants (Abbas et al., 2018; Zemanová et al., 2021). Arsenic limits the efficiency of carbon uptake by plants through affecting metabolic pathways including photosynthesis, transpiration, and respiration (Garg and Singla, 2011). In addition, arsenic reduces antioxidant activity, stomatal conductance, and cell wall thickness, resulting in lipid peroxidation, carbohydrate damage, DNA damage, and chloroplast membrane injury (Abbas et al., 2018; Nahar et al., 2022). Arsenic induces leaf necrosis and leaf senescence, a reduction in leaf number and chlorosis, and eventually leads to defoliation. Additionally, arsenic inhibits plant growth by limiting root proliferation and extension, hence diminishing plant yield and biomass (Finnegan and Chen, 2012; Abbas et al., 2018; Nahar et al., 2022).

Excess of heavy metals not only induces the over-generation of ROS in plants, but also leads to the production of high levels of another compound known as methylglyoxal (MG), which can have detrimental effects on plants (Kumar and Yadav, 2009). These two compounds (ROS and MG) are produced by plants when they are subjected to stressful conditions, and they in excess can disturb plant metabolism and cause cell membrane damage (Yadav et al., 2005a). This has been reported by numerous researchers (Namdjoyan and Kermanian, 2013; Mousavi et al., 2020). Plants have several defense mechanisms for scavenging ROS, including the activation of both antioxidant and non-enzymatic antioxidants’ activity. The most important antioxidant enzymes include superoxide dismutase (SOD), glutathione reductase (GR), peroxidase (POD), ascorbate peroxidase (APX), catalase (CAT), monode-hydroascorbate reductase (MDHAR), and dehydroascorbate reductase (DHAR) (Gill and Tuteja, 2010; Sarker and Oba, 2018). The most important non-enzymatic antioxidants include phenolic compounds, tocopherols, glutathione (GSH), and ascorbic acid (AsA) (Ahmad et al., 2010; Das and Roychoudhury, 2014). On the other hand, the glyoxalase system is one of the primary defense mechanisms in MG scavenging, consisting of a number of enzymes, such as glyoxalase Gly I and Gly II (Hasanuzzaman et al., 2011a; Hasanuzzaman et al., 2017). Therefore, the antioxidant capacity and the glyoxalase system are the primary defense mechanisms of plants against ROS and MG, which can improve plant tolerance against various stressors including As toxicity.

In recent decades, NPs have been used as environmentally safe and eco-friendly materials to reduce or eliminate environmental contamination (Guerra et al., 2018; Sanaeimehr et al., 2018). Zinc oxide nanoparticles (ZnO-NPs) with strong physical adsorption and chemical catalytic capabilities have been identified as a cost-effective solution for environmental remediation (Raja et al., 2018). ZnO-NPs with a small size, intense solubility, and specific surface area can enhance zinc (Zn) absorption in plants. This eliminates the plant’s zinc shortage (Rastogi et al., 2019). ZnO-NPs have been demonstrated to promote growth and development in numerous plant species (Rizwan et al., 2019a; Faizan et al., 2021a; Faizan et al., 2021b). In addition, it has been shown that ZnO-NPs play an effective role in increasing antioxidants capacity, decreasing ROS compounds, and increasing secondary metabolites such as phenolic compounds (Babajani et al., 2019; Ahmad et al., 2020; Faizan et al., 2021a). TiO_2_ NPs are another functional compounds that are employed to protect plants from stress conditions (Mohammadi and Maali-Amiri, 2014). Recent studies have demonstrated that TiO_2_NPs can boost plant growth under stressful conditions (Faraji and Sepehri, 2018; Emamverdian et al., 2021a; Emamverdian et al., 2021b). TiO_2_ reduces the toxicity of cadmium on green algae (*Chlamydomonas reinhardtii* P.A. Dang) (Yang et al., 2012) as well as on soybeans (Singh and Lee, 2016). Our previous research demonstrated that TiO_2_NPs significantly reduced Pb and Cd levels in bamboo species (Emamverdian et al., 2021a; Emamverdian et al., 2021b). However, some evidence suggests that TiO_2_ NPs can inhibit plant metabolism (Khodakovskaya et al., 2012; Li et al., 2012). This indicates that the effectiveness of NPs can vary based on plant species, NPs type, size, and concentration, as well as the presence of heavy metals (Gao et al., 2013). SiO_2_ NPs are essential types of nanomaterials that can have beneficial effects on plant growth under stressful conditions (Slomberg and Schoenfisch, 2012; Siddiqui and Al-Whaibi, 2014). External and internal mechanisms are involved in the toxicity reduction of heavy metals in plants by SiO_2_NPs. The internal mechanisms include the control of metal ion transport *via* the plasma membrane by an alternation in cell wall composition; the synthesis of metal complexes; the enhancement of vacuolar compartmentalization of metal ions; and the enhancement of antioxidant enzyme capacity. The external mechanism is related to the function of SiO_2_NPs in restricting uptake and absorbance of metal ions on the plant root surface (Tubaña and Heckman, 2015). According to a prior study, ZnO NPs, TiO_2_NPs, and SiO_2_ NPs are the optimal choices for reducing plant stress and increasing plant tolerance. The novelty of this work lies in the fact that three distinct nanoparticles in single and combination forms were tested on *Pleioblastus pygmaeus* under two different arsenic levels, which was the first study of this kind on a bamboo plant. This can increase the safety of bamboo as an economic and nutrient source for local populations in China and South Asia. Also, this study makes it possible to evaluate the use of bamboo in phytoremediation technology and pollution removal by providing a foundational understanding of the mechanisms involved. Due to its large biomass, high yields and rapid vegetative growth, bamboo is an excellent candidate for use in phytoremediation technologies (Bian et al., 2020) and can be distinctive among hyper-accumulator plants. On the other hand, ornamental plants have demonstrated a great resistance to metal toxicity and high remedial capacity, making them appropriate for phytoremediation purposes (González-Chávez and Carrillo-González, 2013).

The bamboo species *P. pygmaeus* is commonly utilized for landscaping purposes. This ornamental species of bamboo plant with a height and length of between 30 cm and 50 cm was initially transferred from Japan to China in the 20th century, and it can thrive in a variety of soil types, including acidic, basic (alkaline), and neutral soils (Huang et al., 2020; Huang et al., 2022). In addition, it is planted across a vast area of the Chinese mainland, including Jiangsu province. Today, heavy metals have become a growing issue of concern in the south and southwest of China (Emamverdian et al., 2021a; Emamverdian et al., 2021b). In an investigation on edible plants, several local marketplaces were surveyed in scores of Chinese cities. The results indicated that bamboo shoots contained high amounts of As (Zhao et al., 2006), which clearly demonstrates the infiltration of this hazardous element into human body by contaminating vast areas of the bamboo forests and their consumption by a large population in China. The aim of this study was to investigate how three distinct nanoparticles individually or in combination can influence the bamboo plant`s key enzymatic and non-enzymatic antioxidants, glyoxalase defense system along with some photosynthetic and growth attributes and potentially help the plant overcome some of the negative impacts arising from excess arsenic conditions. We hypothesized that a combination of three NPs may substantially increase plant tolerance to As toxicity by boosting antioxidant and glyoxalase defense systems, phytochelatins metabolism, and reducing translocation and accumulation of As in plant organs.

## 2 Materials and methods

### 2.1 *In vitro* conditions and supplied material

For *in vitro* experiments, ten mm-long nodal bamboo shoots were obtained from one-year-old branches of a single clone of bamboo species (*P. pygmaeus*), which were used for the plant tissue culture in a solid MS medium (Murashige and Skoog, 1962) containing 100 mL macronutrients, 4 mL 6-benzylaminopurine (6-BAP), 0.5 mL kinetin (KT), 10 mL micronutrients, 10 g agar, 25 g sucrose, and a pH of 5.8 ± 0.1. Bamboo roots proliferated from the bamboo shoots. An MS medium containing 1.2 μM thiamine–HCl, 3 μM pyridoxine, 4 μM nicotinic acid, 0.6 mM myoinositol, 0.1 mg L^−1^indole-3-acetic acid (IAA), and 25 g L^−1^ sucrose was prepared. Then, the pre-determined quantities of the experimental treatments were prepared, i.e. 150 μM ZnO NPs, 150 μM TiO_2_ NPs, and 150 μM SiO_2_ NPs in combination and individual forms with 150 and 250 μM As. Also, 9–12 g/L agar was added to 1 L of the MS medium with a pH value of 5.8 ± 0.1. Then, the mixture was transferred to glass petri dishes with a diameter of 60 mm and a volume of 100 mL, and sanitized in an autoclave machine (HiClave HVE-50, Zealway, Delaware, USA) at 120°C for 27 minutes. Next, the petri dishes were placed beneath an incubation hood (Air Tech). Then, three young bamboo shoots were cultured in a medium in four replications per treatment. The Air Tech hood included fluorescent white bulbs with wavelengths of 10 and 420 nm. The medium was sterilized with ultraviolet light for 4 hours at a temperature of 30°C. Finally, the samples were transferred to a tissue culture chamber room under controlled conditions (65% to 90% humidity), which was illuminated by white fluorescent lights with a wavelength range of 10 to 420 nm, a photoperiod of 16 h, and a diurnal and nocturnal temperature range of 30/25°C and 17/22°C for three weeks, respectively.

The treatments consisted of 150 μM ZnO NPs, 150 μM TiO_2_ NPs, and 150 μM SiO_2_ NPs, as well as 150 μM and 250 μM As, individually and in combination. The concentration of nanoparticles and arsenic was determined based on our prior research work in which the low and high levels of NPs and As were determined within the tolerance range of the bamboo species.

NPs (ZnO NPs, TiO_2_ NPs, and SiO_2_NPs) were spherical powders with a 25 nm diameter and a purity of 95-99%, which were supplied by a local company (Nanjing Jiancheng) in Jiangsu Province, China.

The bamboo species (*P. pygmaeus*) had been selected from the region’s species that are grown at the bamboo garden of the bamboo research Institute of Nanjing Forestry University (Nanjing, Jiangsu, China)

### 2.2 Determination of As content

In this study, the As content of the bamboo plant organs including stems, roots, and leaves was determined. First, following cleaning, the samples were transferred to an oven to dry. The samples were then homogenized with 70% nitric acid and stored at 75°C for 18 minutes before being centrifuged at 8000 × *g* for 30 minutes. The As content in the stems, roots, and leaves was determined and then analyzed using an atomic absorption spectrophotometer (High-Tech). A graphite furnace with Zeeman-effect background correction (AAnalyst 800, Perkin Elmer, Norwalk, CT, USA) was fitted to the spectrophotometer. For the standardization of metals, 2.5% nitric acid was utilized as a wash solvent in “spectra scan” mode. In unattended automated analysis, standard calibration and verification (Perkin Elmer) for all elements on the mineral target analyzer (TAL) list were performed at appropriate intervals.

### 2.3 Determination of glutathione, ascorbic acid, and phytochelatins content

Fresh roots (0.5 g) were homogenized in 3 mL ice-cold 5% metaphosphoric acid containing 1 mM EDTA, and then centrifuged at 9,000 ×*g* for 30 minutes. The obtained supernatant was used to determine the GSH and AsA contents.

The analysis of GSH was performed using Gill’s protocol (Gill et al., 2015). The reaction mixture (1 mL) comprising of 6 mM DTNB, 0.3 mM NADPH, and 10 U mL^–1^ glutathione reductase (GR) was added to 200 µl of the supernatant for the measurement of total glutathione (GSSG + GSH). The supernatant was then incubated at 30°C for 25 minutes with 2-vinylpyridine and triethanolamine (50%, v/v). An absorbance range of 412 nm was utilized to record the content of GSSG’s composition. The GSH content was obtained by subtracting the GSSG content from the total glutathione using the Gill, et al. (Gill et al., 2015) method.

For measuring AsA content, 200 µl of the obtained supernatants were neutralized with a 0.5 M K-P buffer at a pH of 7.0, and then 0.1 M dithiothreitol was added to reduce the amount of oxidized AsA in the extract. After adding 100 mM K-P buffer with pH=7 and 1 unit of ascorbate oxidase to the samples, the total absorbance of AsA was determined to be 265 nm using the standard curve of AsA (Charest and Ton Phan, 1990). Finally, oxidized AsA (DHA) was calculated by using the formula below:


(1) Oxidized AsA=AsA(total)–reduced AsA

The non-protein thiols extraction was utilized to assess the concentration of phytochelatins by using the method developed by De Vos, et al. (De Vos et al., 1992). To this end, 4 mL of sulfosalicylic acid (3%) was homogenized in 0.5 g bamboo root and leaf treatments. Then, it was centrifuged at 10.000 ×*g* at 5°C for 17 minutes and then was added to a reaction mixture containing 0.6 mM 5,5′-dithiobis (2-nitrobenzoic acid), 5 mM EDTA, and 120 mM of phosphate buffer with a pH value of 7.5. The phytochelatins content was measured by subtracting the GSH obtained by non-protein thiols from the absorbance reading at 412 nm.

### 2.4 Determination of soluble sugars and proline

Proline content was measured by the (Bates et al., 1973) protocol in which 3% sulfosalicylic acid was utilized to homogenize 0.5 mL of leaf samples. The resulting solution was centrifuged for 25 minutes at a range of 10.000 ×*g*. The supernatant was then combined with 2 mL acid ninhydrin and 1% glacial acetic acid and incubated for 3 hours at 100°C. The mixture was cooled in an ice bath before toluene was added. Proline content was determined by measuring the absorbance at 520 nm, by comparison with the standard curve. The analysis of soluble sugars was performed using both the method of (Shi et al., 2015) and the primary methodology. In this process, 0.5 g bamboo leaves were homogenized in 80% ethanol for 20 minutes at 90°C, then it was mixed into the solution, and the extract was boiled for 17 minutes. The acquired absorbance was measured at 630 nm, and the final data were computed according to the sucrose standard curve.

### 2.5 Determination of malondialdehyde, hydrogen peroxide, superoxide radical, and electrolyte leakage

The H_2_O_2_ content was measured using the protocol by (Patterson et al., 1984). In these measurements, 0.5 g of leaf samples were homogenized in 10 mL of cold acetone in a mortar and pestle. The sample was then centrifuged at 4500× *g* for 20 minutes. Next, 1 mL of supernatant was added to the mixture consisting of 2 mL of ammonia (17 M), 2 mL of HCl, and 2 mL of 20% titanium chloride. For proper absorbance, the supernatant was extracted with 2 N H_2_SO_4_ in 10 mL using acetone. Again, the mixture was centrifuged to eliminate immiscible components. The absorbance was then measured at 410 nm. The levels of H_2_O_2_ were determined using a standard curve and expressed in μmole g^−1^ FM.

The superoxide radical ( ${\text{O}}_{2}^{•-}$ ) was measured by using the method of (Li et al., 2010). In this process, 200 mg of leaf tissue samples were homogenized with phosphate buffer (65 mM- pH = 7.8) and centrifuged at 4500× *g* for 17 minutes. The remaining supernatant was added to phosphate buffer (65 mM) with a pH of 7.8 and hydroxylamine hydrochloride (10 mM) at 32 °C for 25 minutes. Then, 17 mM of sulfanilamide and 7 mM of α-naphthylamine were added to the solution, which was kept at 30 °C for 25 minutes while the absorbance at 530 nm was measured.

MDA was determined using the (Siddique et al., 2012) method. According to this method, 0.5 g of fresh leaves were added to 0.1% trichloroacetic acid (TCA) that was centrifuged at the optimal 7000× *g* for 30 minutes. The obtained amount of supernatant was resolved in a 20% TCA solution containing 5% thiobarbituric acid, and preserved at 99 °C for 20 minutes, which was centrifuged again at 2000× *g* for 20 minutes at 4 °C for the second time. Absorbance measurements revealed the presence of malondialdehyde content at 532 nm.

Electrolyte leakage (EL) was measured using the (Valentovic et al., 2006) method. In the first step, 15 mL of deionized water was added to 0.3 g leaf, followed by 2.5 hours of storage at 28°C during which the initial electrical conductivity of the solution, EC_1_, was recorded. For the determination of EC_2_ or secondary electrical conductivity, the treatments were transferred and preserved in an autoclave at 110°C for 25 minutes, and EL was determined using the below formula.


(2) $$\text{EL}\left(\%\right)={\text{EC}}_{1}/{\text{EC}}_{2}\times 100$$

### 2.6 Estimation of antioxidant enzymes activity and glyoxalase defense system

In the initial preparatory step for enzyme experiment, 0.5 g of fresh leaves were rinsed and cleaned before being cut by scissors and placed in a pestle. Liquid nitrogen (LN) was then combined with the leaves and then the mixture was pulverized. The resulting powder was then mixed with 2 mg phosphate-buffered saline with a pH range between 7.2–7.4 and maintained between 2–8°C, followed by 20 minutes of centrifugation between 2500–3500× *g*. Antioxidants were extracted from the supernatant.

Superoxide dismutase (SOD) activity was measured using the (Dhindsa and Matowe, 1981) method. In this process, SOD was extracted using the reduction of photochemically produced nitro blue tetrazolium (NBT), whose absorbance was measured at 560 nm. The activity of peroxidase (POD) was determined based on the (Zhang, 1992) methodology, in which the POD concentration was measured using absorbance alternation at 470 nm. Catalase (CAT) activity was measured using the (Aebi, 1984) method by measuring absorbance at 240 nm. The glutathione reductase (GR) was evaluated using the (Foster and Hess, 1980) method, and its absorbance was measured at 340 nm, resulting in a value of EU mg^−1^ protein. The activity of ascorbate peroxidase (APX) was measured using the (Nakano and Asada, 1981), with the absorbance recorded at 290 nm and defined as EU mg^−1^ protein. Glyoxalase (Gly) activity was measured using the (Hasanuzzaman et al., 2011a) method. In this method, the reaction included 3.5 mM MG, a potassium-phosphate buffer (100 mM) with a pH range of 7.0, magnesium sulfate (15 mM), and 1.7 mM GSH. The absorbance at 240 nm was measured for 1 minute. The Gly II activity was measured using (Principato et al., 1987) method. In this method, a solution containing Tris–HCl buffer (100 mM) with a pH value of 7.0, 1 mM S-D-lactoylglutathione, 0.2 mM 5,5´-dithiobis (2-nitrobenzoic acid) (DTNB), and protein extract at 412 nm for 1 min was recorded. Methylglyoxal (MG) was measured using the Yadav et al., (2005b) method. For this purpose, 100 μL of 5 M perchloric acid, 1 mL of total reaction volume, 250 μL of 7.2 mM 1, 2-diaminobenzene, and 650 μL of the supernatant were combined. The acquired absorbance of the derivatized MG was recorded in the absorbance range of 200–500 nm for 17 cycles separated by 1 minute.

### 2.7 Photosynthetic pigments, gas exchange parameters and chlorophyll fluorescence

The photosynthesis parameters (chlorophyll a (Chl a), chlorophyll b (Chl b), carotenoids) were determined using the (Lichtenthaler and Buschmann, 2001) method (using a solution by 0.5 g of bamboo leaf + 20 ml 80% acetone as the extraction solvent). In this method, Chl a, Chl b, and carotenoid levels were measured using at an absorbance rate of 663, 645, and 470 nm, respectively, which was ultimately computed using the following formulas:


(3) Chl a=12.25∗(A663)−2.79∗(A647)

(4) Chl b=21.50∗(A647)−5.10∗(A663)

(5) Total Chlorophyll=(Chl a)+(Chl b)

(6) Carotenoids=1000∗(A470)–1.82∗Chl a–95.15∗Chl b/225

The unit of calculation was mg/g fresh weight.

The parameters related to leaf gas exchange and chlorophyll fluorescence were measured using a portable gas exchange system (GFS-3000; Walz) and a PAM fluorometer (Walz; PAM 2500) between 7 and 9 a.m.

### 2.8 Shoot, root dry weight and plant height

This study determined plant biomass indicators such as root dry weight, shoot dry weight, and plant height (shoot length). To do this, the samples were first cleaned and then placed in an oven to eliminate the surface moisture. The temperature was then maintained at 110°C for 30 minutes. The plant biomass (roots and shoots dry weight) was determined by keeping the treatments at 60°C for 15 hours, and then each sample was weighed individually. The plant height was determined by comparing bamboo shoots at the beginning and end of the experiment.

### 2.9 Measuring the bioaccumulation factor, tolerance index, and translocation factor

To investigate plant tolerance to arsenic toxicity, three indices, namely the bioaccumulation factor (BAF), the translocation factor (TF), and the tolerance index (TI), were determined using the (Souri and Karimi, 2017) method, which highlights the efficiency of phytoextraction. Consequently, these indexes were computed using the following formula:


$$\begin{array}{c} \text{Translocation factor in leaves and stem}\\ =\text{the content of As in leaves and stem}\left(\text{mg}/\text{kg}\right)/\text{the content of As in roots} \end{array}$$

$$\begin{array}{c} \text{Tolerance index in shoot}/\text{root}\\ =\text{dry weight of plant shoot}/\text{root in treatments}\left(\text{g}\right)/\text{dry weight of plant shoot/root in the control}\left(\text{g}\right) \end{array}$$

$$\begin{array}{c} \text{Leaves bioaccumulation factor}\\ =\text{the content of As in the leaves}/\text{the content of As in the medium} \end{array}$$

### 2.10 Statistical analysis

In this experiment, R software was used to perform an analysis of variance (ANOVA) on the data. The experiment design was performed in a completely randomized design (CRD) with a factorial (2-way) and four replications. In addition, mean differences were determined using Tukey’s test with a probability level of p< 0.05.

## 3 Results

### 3.1 Nanoparticles reduce As content in stems, roots, and leaves

In this research, there was a significant difference between different concentrations of NPs alone and in combination with 150 µM and 250 µM As (p<0.001). The use of NPs considerably lowered the As accumulation in the plant organs (root, shoot, and leaves). The greatest reduction of As was observed with the combination of three NPs (ZnO, SiO_2_, and TiO_2_) + 150 µM and 250 µM As where As decrease of 75% and 68% in leaves, 66% and 40% in stems, and 59% and 59% in roots occurred, respectively, compared to the control treatments (**Table 1**). On the other hand, the data analyses demonstrated that the bamboo roots accumulated the highest quantities of As compared to the stems and leaves, indicating that NPs restricted the accumulation of As in the aerial segments of the plants, including leaves and stems, and, therefore, increased the plant tolerance to As toxicity levels.

**Table 1:** The impact of NPs alone and in combination on As accumulation in one-year-old *Pleioblastus pygmaeus*.

NPs concentrations	As levels	As accumulation (leaves)	As accumulation (stem)	As accumulation (root)
µmol l^−1^	µmol l^−1^	µg l^−1^	µg l^−1^	µg l^−1^
0	0	0	0	0
0	150 µM As	44.10± 1.04^m^	52.10± 0.80^l^	63.99± 0.99^m^
0	250 µM As	48.40± 0.72^n^	56.23± 0.95^m^	69.30± 0.99^n^
150 µM Si	0	0	0	0
150 µM Si	150 µM As	30.20± 0.77^h^	35.44 ± 0.72^g^	42.33± 0.97^g^
150 µM Si	250 µM As	41.50± 0.90^l^	50.33± 0.85^kl^	58.65± 1.07^k^
150 µM Ti	0	0	0	0
150 µM Ti	150 µM As	31.22± 0.91^hi^	38.55± 0.82^h^	45.88± 0.82^h^
150 µM Ti	250 µM As	43.37 ± 1.04^lm^	51.22 ± 0.77^kl^	61.21± 0.88^l^
150 µM Zn	0	0	0	0
150 µM Zn	150 µM As	27.56± 0.79^g^	32.44± 1.08^f^	40.10± 1.12^f^
150 µM Zn	250 µM As	39.10± 0.93^k^	49.33± 0.65^k^	56.77± 0.80^k^
150 µM Si+Ti	0	0	0	0
150 µM Si+Ti	150 µM As	24.30± 0.82^f^	29.43± 0.70^e^	36.41± 0.66^e^
150 µM Si+Ti	250 µM As	38.20± 0.84^k^	46.66± 0.96^j^	52.43± 1.01^j^
150 µM Zn+Si	0	0	0	0
150 µM Zn+Si	150 µM As	18.33 ± 0.77^d^	24.44 ± 0.77^d^	31.30± 0.88^d^
150 µM Zn+Si	250 µM As	33.10 ± 0.80^i^	39.88 ± 0.89^h^	48.66± 1.05^i^
150 µM Zn+Ti	0	0	0	0
150 µM Zn+Ti	150 µM As	21.40± 0.89^e^	27.55± 1.10^e^	34.56± 0.84^e^
150 µM Zn+Ti	250 µM As	36.11 ± 1.03^j^	42.77 ± 1.02^i^	51.21± 0.87^j^
150 µM Si+Ti+Zn	0	0	0	0
150 µM Si+Ti+Zn	150 µM As	11.31± 0.90^b^	17.30± 0.97^b^	25.70± 0.89^b^
150 µM Si+Ti+Zn	250 µM As	15.40± 1.06^c^	22.44± 0.82^c^	28.40± 1.01^c^

In this table, different letter(s) indicated significant differences following the application of NPs with 150 and 250 µM As based on Tukey′s test (p< 0.05).

### 3.2 Calculation of translocation factor, bioaccumulation factor, and tolerance index

According to the calculation results of TF, BAF, and TI, there was a significant difference between NPs levels alone and in combination with As (p<0.001). Hence, the greatest plant tolerance under As occurred with the treatments involving the combination of ZnO +SiO_2_+ TiO_2_ with 150 µM and 250 µM As where an increase of 29% and 24% in the TI of the shoot and 25% and 14% in the TI of the root were respectively observed. The results of this study demonstrated that NPs considerably inhibited the transport of As from the roots to the stems and leaves, resulting in a reduction of As accumulation in the stems and leaves. This was shown in the treatment consisting of the combined application of three NPs, which gave the lowest amount of BAF in the bamboo leaves under 150 µM and 250 µM As with 75% and 68% reduction in comparison with their control treatments (**Table 2**).

**Table 2:** Changes in the translocation factor (TF) of bamboo leaves and stem, tolerance index (TI) of shoot and root, as well as bioaccumulation factor (BF) of leaves in response to 150 µM NPs in combination or single form when exposed to 150 µM and 250 µM As, compared to the control treatment.

Treatments	TF (leaves)	TF (stem)	TI (shoot)	TI (root)	BF (leaves)
0	0.00 ± 0.00^a^	0.00 ± 0.00^a^	1.00 ± 0.00^kl^	1.00 ± 0.00^g-k^	0.00 ± 0.00^f^
150 µM As	0.68 ± 0.01^ef^	0.81 ± 0.01^cde^	0.58± 0.02^ab^	0.68 ± 0.05^ab^	0.29 ± 0.00^de^
250 µM As	0.69 ± 0.01^g^	0.81± 0.00^cde^	0.54± 0.07^a^	0.64 ± 0.02^a^	0.19 ± 0.00^bcde^
150 µM Si	0.00 ± 0.00^a^	0.00 ± 0.00^a^	1.09 ± 0.01^mn^	1.08± 0.05^i-m^	0.00 ± 0.00^f^
150 µM Si + 150 µM As	0.70 ± 0.05^fg^	0.83± 0.01^defg^	0.76± 0.04^fgh^	0.88 ± 0.02^c-h^	0.20± 0.22^cde^
150 µM Si + 250 µM As	0.70 ± 0.03^fg^	0.85 ± 0.00^fgh^	0.61 ± 0.01^abcd^	0.70± 0.00^abc^	0.16± 0.00^abc^
150 µM Ti	0.00 ± 0.00^a^	0.00 ± 0.00^a^	1.05 ± 0.04^lm^	1.04 ± 0.03^h-l^	0.00 ± 0.00^f^
150 µM Ti + 150 µM As	0.68 ± 0.05^ef^	0.84 ± 0.00^efg^	0.74± 0.00^fg^	0.85 ± 0.07^b-g^	0.20± 0.00^cde^
150 µM Ti + 250 µM As	0.70 ± 0.03^fg^	0.83± 0.00^defg^	0.60± 0.00^abc^	0.68± 0.00^ab^	0.17 ± 0.00^abcd^
150 µM Zn	0.00 ± 0.00^a^	0.00 ± 0.00^a^	1.14± 0.05^no^	1.12 ± 0.12^j-n^	0.00 ± 0.00^f^
150 µM Zn +150 µM As	0.68 ± 0.06^ef^	0.80± 0.04^cde^	0.79 ± 0.00^ghi^	0.91 ± 0.12^d-i^	0.18± 0.00^abcd^
150 µM Zn + 250 µM As	0.68 ± 0.01^ef^	0.86± 0.00^gh^	0.63± 0.00^bcd^	0.74± 0.04^abcd^	0.15± 0.00^abc^
150 µM Si+Ti	0.00 ± 0.00^a^	0.00 ± 0.00^a^	1.19 ± 0.02^bop^	1.13± 0.04^k-n^	0.00 ± 0.00^f^
150 µM Si+Ti+150 µM As	0.66 ± 0.01^def^	0.80 ± 0.00^cde^	0.82 ± 0.01^hi^	0.93 ± 0.06^e-i^	0.16 ± 0.00^abc^
150 µM Si+Ti+ 250 µM As	0.72 ± 0.04^gh^	0.88± 0.00^h^	0.66 ± 0.01^cde^	0.75 ± 0.01^abcd^	0.15 ± 0.00^abc^
150 µM Zn+Si	0.00 ± 0.00^a^	0.00 ± 0.00^a^	1.27 ± 0.06^qr^	1.24± 0.16^mn^	0.00 ± 0.00^f^
150 µM Zn+Si + 150 µM As	0.58 ± 0.05^cd^	0.78± 0.00^c^	0.94± 0.00^jk^	0.97 ± 0.02^f-k^	0.12 ± 0.00^abc^
150 µM Zn+Si + 250 µM As	0.68 ± 0.02^ef^	0.81± 0.00^cdef^	0.72± 0.01_efg_	0.81 ± 0.03^a-f^	0.13± 0.00^abc^
150 µM Zn+Ti	0.00 ± 0.00^a^	0.00 ± 0.00^a^	1.27± 0.00^pqr^	1.20 ± 0.06^lmn^	0.00 ± 0.00^f^
150 µM Zn+Ti + 150 µM As	0.61 ± 0.04^cde^	0.79 ± 0.01^cd^	0.87± 0.00^ij^	0.95 ± 0.03^e-j^	0.14 ± 0.00^abc^
150 µM Zn+Ti + 250 µM As	0.70 ± 0.03^fg^	0.83± 0.00^defg^	0.68± 0.01^def^	0.79± 0.01^a-e^	0.14 ± 0.00^abc^
150 µM Si+Ti+Zn	0.00 ± 0.00^a^	0.00 ± 0.00^a^	1.32± 0.01^r^	1.26± 0.07^n^	0.00 ± 0.00^f^
150 µM Si+Ti+Zn +150 µM As	0.44 ± 0.03^b^	0.67± 0.02^b^	1.29 ± 0.02^qr^	1.25 ± 0.08^mn^	0.07 ± 0.00^ab^
150 µM Si+Ti+Zn + 250 µM As	0.54 ± 0.04^c^	0.79± 0.04^cd^	1.24± 0.02^pq^	1.14 ± 0.04^k-n^	0.06± 0.00^a^

Each data point is the mean ± standard error for four replicates. The letters a, b, c, etc. indicated significant differences following the application of NPs with 150 and 250 uM As based on Turkey's test(p< 0.05).

### 3.3 Nanoparticles increase glutathione, ascorbic acid, and phytochelatins content

**Figure 1:**
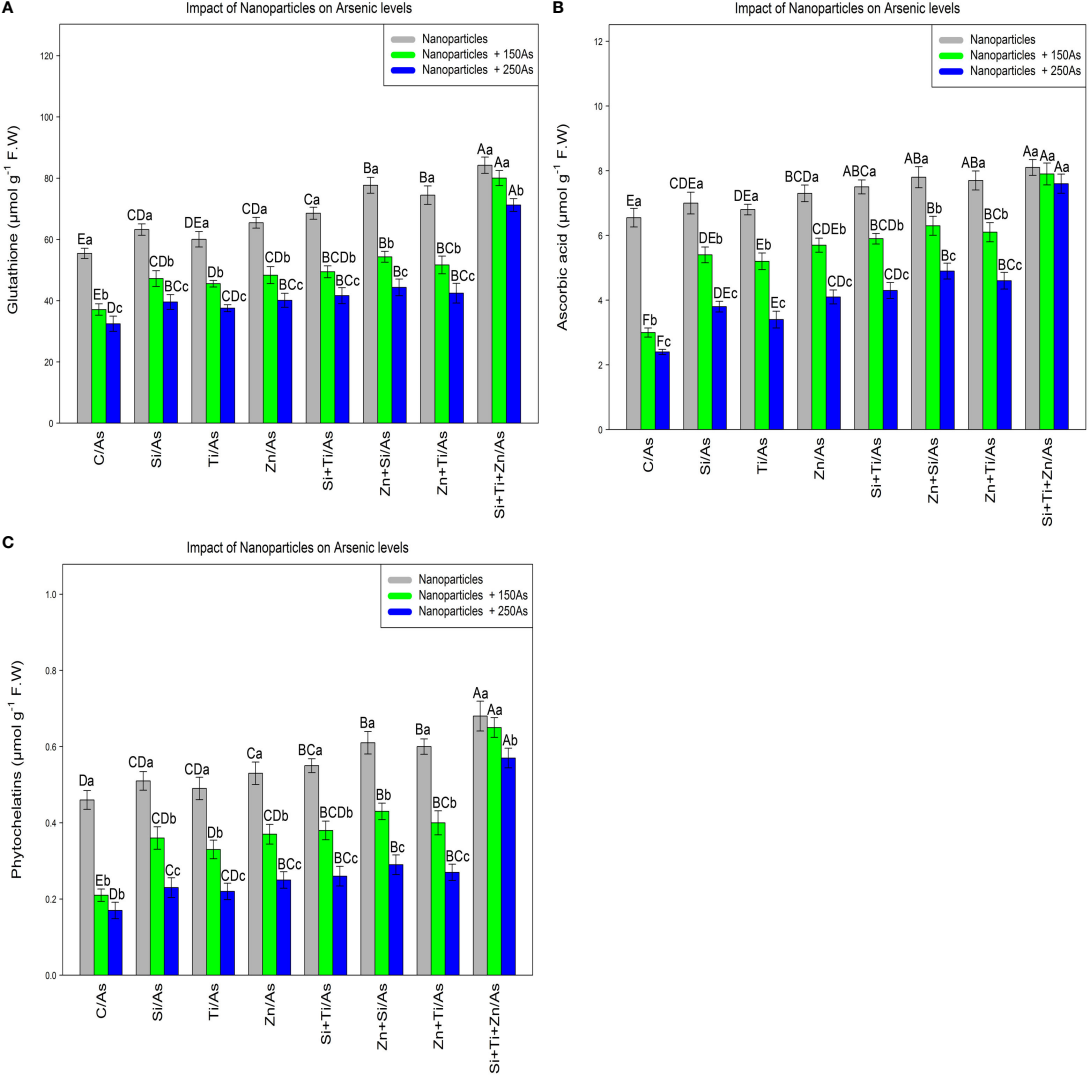
The impact of NPs alone and in combination on the concentration of glutathione **(A)**, ascorbic acid **(B)** and phytochelatins **(C)** in one-year-old *Pleioblastus pygmaeus* exposed to 150 and 250 µM As (four replicates). In this figure, the letters a, b, c, *etc.* indicated significant differences following the application of NPs with 150 and 250 µM As based on Tukey′s test (p< 0.05).

In this experiment, the glutathione, ascorbic acid, and phytochelatins contents were measured to evaluate the efficacy of plant defense mechanisms in response to different types of arsenic toxicity. The data analyses demonstrated a significant difference between the various types of NPs alone and in combination with 150 µm and 250 µM As for glutathione, ascorbic acid, and phytochelatins contents (p<0.001) (**Figure 1**). Based on the results, the addition of NPs significantly increased GSH, ascorbic acid, and phytochelatins contents. The greatest increase was attributed to the combination of three types of NPs and As at 150 and 250 µM with respective increases of 51%, 44%, and 28% in GSH, 23%, 20%, and 16% in ascorbic acid, and 47%, 41%, and 24% in phytochelatins content in comparison to the control treatments. This revealed that the combination of three NPs was the most effective treatment for enhancing the plant defense mechanism, as measured by an increase in the GSH, ascorbic acid, and phytochelatins contents under 150 and 250 µM As. Meanwhile, the combination of (ZnO and SiO_2_) NPs and (ZnO –TiO_2_) NPs remarkably enhanced the GSH, ascorbic acid, and phytochelatins contents in the bamboo plant under 250 µM As.

### 3.4 Nanoparticles raise soluble sugars and proline content under As toxicity

**Figure 2:**
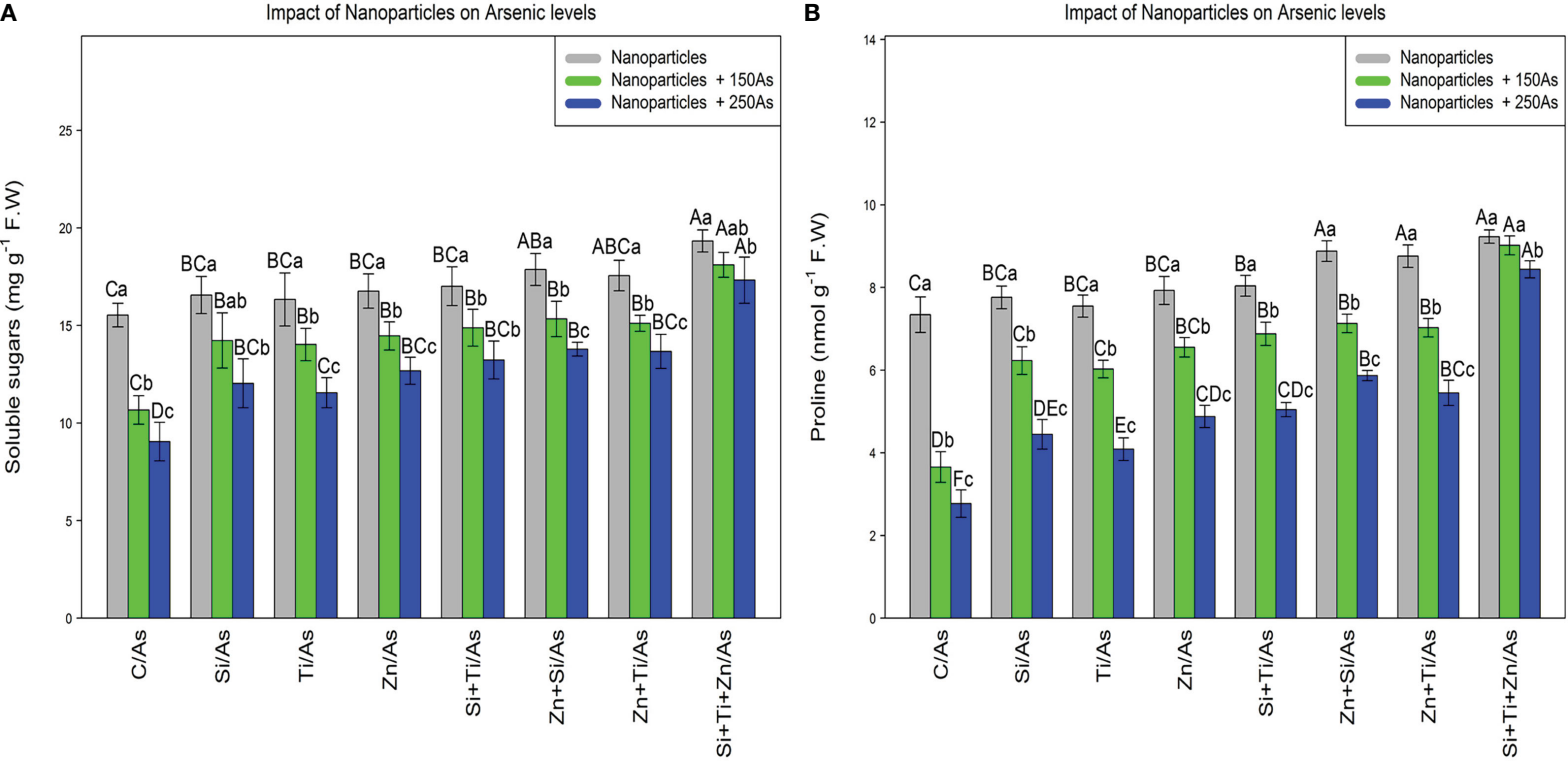
The impact of NPs alone and in combination on the concentration of soluble sugars **(A)**, and proline **(B)** in one-year-old *Pleioblastus pygmaeus* exposed to 150 and 250 µM As (four replicates). In this figure, the letters a, b, c, *etc.* indicated significant differences following the application of NPs with 150 and 250 µM As based on Tukey′s test (p< 0.05).

The soluble sugars and proline content were significantly affected by the different levels of NPs and As (P< 0.001). The use of NPs significantly increased the soluble sugars and proline content in the bamboo species. The greatest increase in soluble sugars and proline content was obtained with the combined and single application of (ZnO, SiO_2_, and TiO_2_) NP_S_ at 150 µM As where a 24% and 16% increase in soluble sugars and a 25% and 22% increase in proline content were respectively observed in comparison to the control treatments. NPs, when used combined, significantly enhanced the soluble sugars and proline content in the bamboo exposed to 250 µM As, with an 11% increase in the soluble sugars and a 25% increase in the proline content compared to the control treatment (**Figure 2**).

### 3.5 Nanoparticles reduce ROS compounds and confer protection to cell membranes

**Figure 3:**
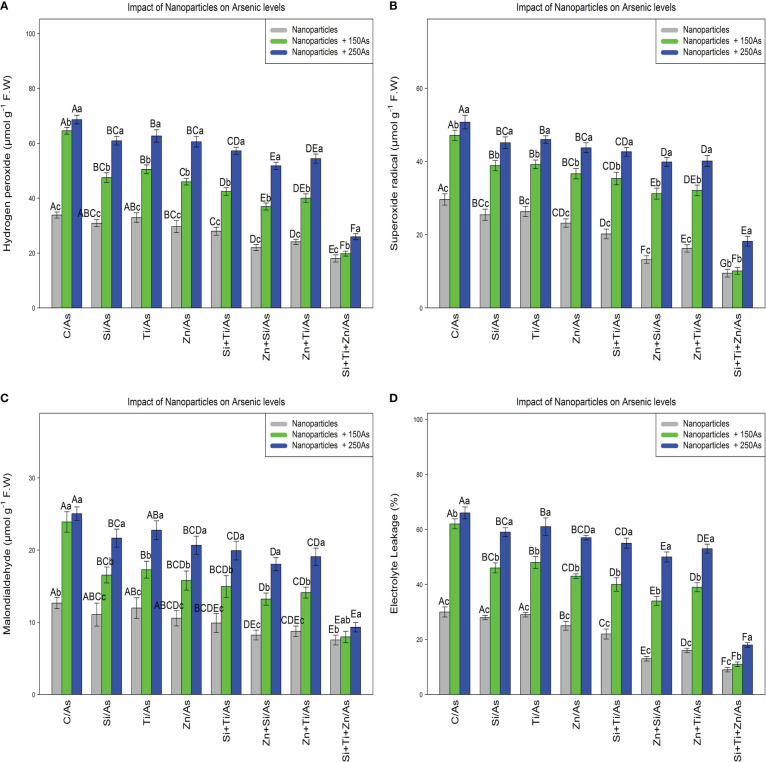
The impact of NPs alone and in combination on hydrogen peroxide **(A)**, superoxide radical **(B)**, malondialdehyde **(C)**, and electrolyte leakage **(D)** in one-year-old *Pleioblastus pygmaeus* exposed to 150 and 250 µM As (four replicates). In this figure, the letters a, b, c, *etc.* indicated significant differences following the application of NPs with 150 and 250 µM As based on Tukey′s test (p< 0.05).

Malondialdehyde (MDA), hydrogen peroxide (H_2_O_2_), superoxide radical ( ${\text{O}}_{2}^{•-}$ ), and electrolyte leakage (EL) were determined to assess the impact of NPs on ROS compounds and plant cell membranes. The results indicated that NPs alone and in combination significantly reduced ROS compounds and the indices values related to cell membrane degradation, with a significant difference between the treatments (P< 0.001) where the lowest amounts were related to the combination of three NPs at 150 µM and 250 µM As, with a 44% and 26% reduction in H_2_O_2_, 65%, and 38% reduction in ${\text{O}}_{2}^{•-}$ , 36% and 26% decrement in MDA, and 63% and 40% decrement in EL compared to the control treatment (**Figure 3**). Also, the results showed that the addition of NPs in single form significantly reduced MDA,H_2_O_2_, ${\text{O}}_{2}^{•-}$ contents as well as percentage of EL in the bamboo plants under 150 µM and 250 µM As. 

### 3.6 Nanoparticles increase antioxidant capacity and glyoxalase defense system

**Figure 4:**
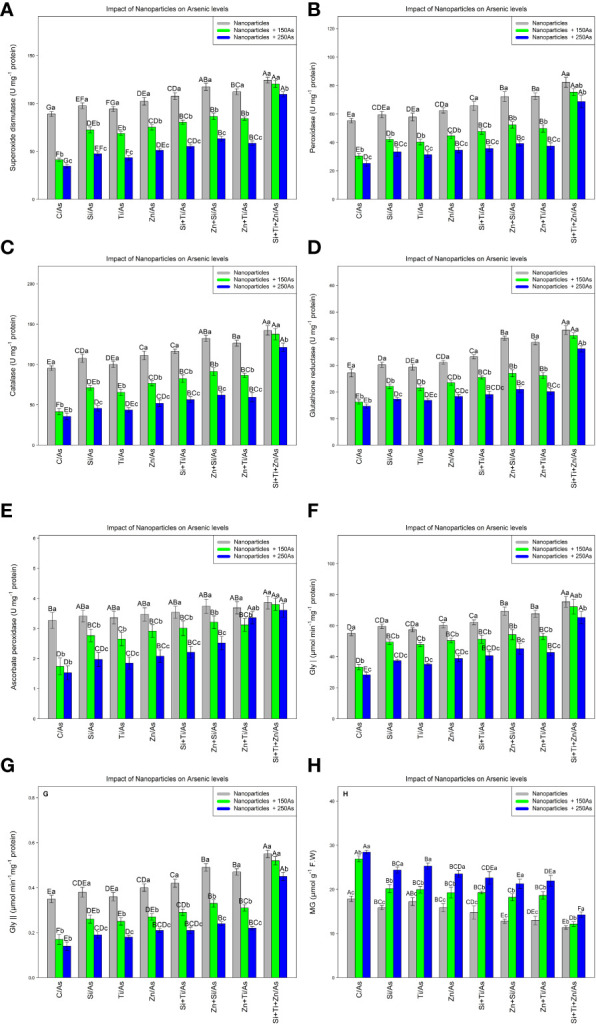
The impact of NPs alone and in combination on superoxide dismutase (SOD) **(A)**, peroxidase (POD) **(B)**, catalase (CAT) **(C)**, glutathione reductase (GR) **(D)**, ascorbate peroxidase (APX) **(E)**, glyoxalase I **(F)**, glyoxalase II **(G)**, and MG **(H)** in one-year-old *Pleioblastus pygmaeus* exposed to 150 and 250 µM As (four replicates). In this figure, the letters a, b, c, *etc.* indicated significant differences following the application of NPs with 150 and 250 µM As based on Tukey′s test (p< 0.05).

The results indicated that NPs containing ZnO, TiO_2_, and SiO_2_ significantly increased antioxidant activity and the glyoxalase defense system, with a significant difference among different treatments (P< 0.001). Hence, the highest antioxidant activity under As toxicity was related to a combination of three NPs, i.e., ZnO, TiO_2_, and SiO_2_ at 150 and 250 µM As where 35% and 23% increases in SOD, 36% and 24% increases in POD, 44% and 27% increases in CAT, %51% and 33% increases in GR, and 16% and 10% increases in APX were respectively observed relative to the control treatments. On the other hand, glyoxalase activity (Gly I and Gly II) was significantly affected by different types of NPs and As concentrations (P< 0.001). NPs significantly increased glyoxalase activity where the greatest rise in Gly I and Gly II activity occurred with a combination of (ZnO, TiO_2_, and SiO_2_) NPs + 150 µM and 250 µM As, respectively, leading to an increase of 31% and 18% in Gly I and 48% and 28% in Gly II compared to the control treatment. The combined application of three NPs resulted in the greatest reduction of MG by 55% and 50% under 150 and 250 µM As, respectively (**Figure 4**).

### 3.7 Nanoparticles improve photosynthetic indices under As toxicity

**Figure 5:**
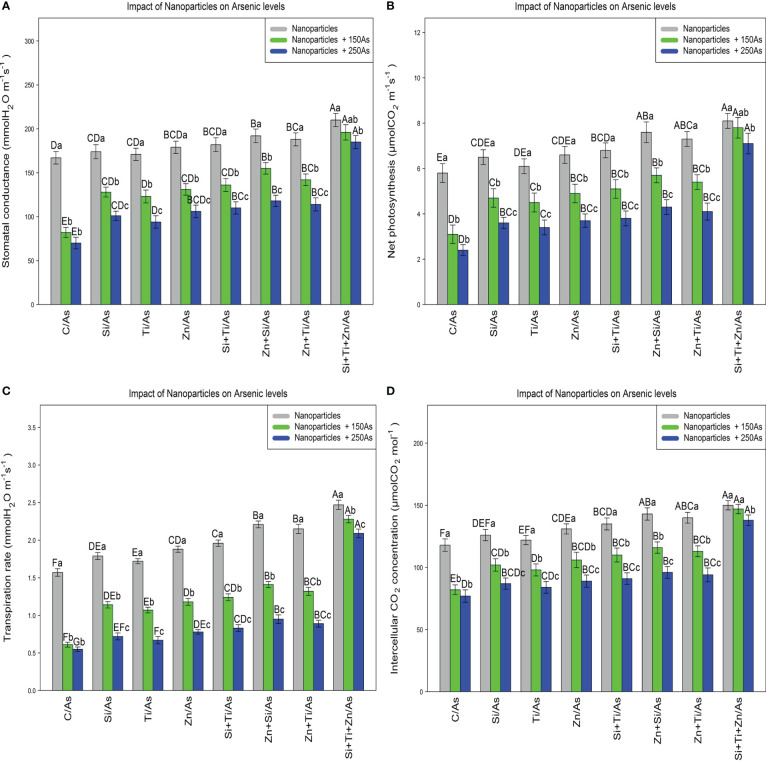
The impact of NPs alone and in combination on stomatal conductance **(A)**, net photosynthesis **(B)**, transpiration rate **(C)**, and concentration of intercellular CO_2_ **(D)** in one-year-old *Pleioblastus pygmaeus* exposed to 150 and 250 µM As (four replicates). In this figure, the letters a, b, c, *etc.* indicated significant differences following the application of NPs with 150 and 250 µM As based on Tukey′s test (p< 0.05).

The results revealed a significant difference in the photosynthetic pigments content by distinct NPs types when the bamboo plant was exposed to 150 and 250 µM As (P<0.001). The greatest increase in the photosynthetic pigments was associated with the combined use of three types of NPs, i.e., ZnO, TiO, SiO_2_, under 150 µM and 250 µM As, with 27% and 19% enhancement in chlorophyll a content, 29% and 19% enhancement in chlorophyll b content, 34% and 25% enhancement in total chlorophyll content, as well as 51% and 34% enhancement in carotenoids content (**Table 3**). Moreover, the results demonstrated that the parameters associated with gas exchange and chlorophyll fluorescence rose significantly when NPs, i.e., ZnO, TiO_2_, and SiO_2_, were combined at 150 µM and 250 µM As (P<0.001). Therefore, the greatest increase in gas exchange parameters and chlorophyll fluorescence was related to the combination of three types of NPs under 150 µM and 250 µM As, with a 17% and 10% increase in stomata conductance, a 34% and 22% increment in net photosynthesis, a 45% and 33% increase in transpiration rate, and 24% and 16% increment in intercellular concentration in comparison to the control treatment, respectively (**Figure 5**).

**Table 3:** The impact of NPs alone and in combination on the photosynthetic pigments (chlorophyll a, chlorophyll b, total chlorophyll and carotenoids) in one-year-old *Pleioblastus pygmaeus*.

Treatment	Chl a	Chl b	Chl (a+b)	Carotenoids
Control	5.67± 0.30^jkl^	7.23± 0.28^ijk^	12.90 ± 0.27^ijkl^	2.01 ± 0.21^ijk^
150 µM As	2.55± 0.18^ab^	3.78± 0.27^ab^	6.33± 0.46^ab^	0.85 ± 0.04^ab^
250 µM As	2.16± 0.18^a^	3.23± 0.24^a^	5.39 ± 0.40^a^	0.65 ± 0.04^a^
150 µM Si	6.02± 0.20^kl^	7.86± 0.24^kl^	13.85 ± 0.40^klmn^	2.17 ± 0.04^kl^
150 µM Si +150 µM As	4.44± 0.17^fgh^	6.03± 0.29^fgh^	10.47± 0.47^efg^	1.41 ± 0.04^efg^
150 µM Si +250 µM As	2.89± 0.14^bc^	4.32± 0.13^bc^	7.21± 0.26^bc^	0.98± 0.08^bc^
150 µM Ti	5.89± 0.27^kl^	7.65± 0.27^jkl^	13.54 ± 0.48^jklm^	2.09 ± 0.06^jk^
150 µM Ti +150 µM As	4.09± 0.27^efg^	5.86± 0.27^efg^	9.95± 0.50^ef^	1.29 ± 0.04^def^
150 µM Ti +250 µM As	2.73± 0.21^ab^	4.06± 0.29^b^	6.79 ± 0.39^abc^	0.91 ± 0.06^b^
150 µM Zn	6.11± 0.21^lm^	8.04± 0.28^lm^	14.15 ± 0.50^lmn^	2.39 ± 0.05^lm^
150 µM Zn +150 µM As	4.76± 0.28^ghi^	6.23± 0.26^fgh^	10.99 ± 0.49^efgh^	1.52 ± 0.06^fg^
150 µM Zn +250 µM As	3.01± 0.27^bc^	4.83± 0.32^cd^	7.84 ± 0.53^bcd^	1.01 ± 0.06^bc^
150 µM Si+Ti	6.34± 0.27^lmn^	8.34± 0.23^lmn^	14.68 ± 0.47^mno^	2.57 ± 0.06^mn^
150 µM Si+Ti +150 µM As	4.98± 0.32^hij^	6.55± 0.25^ghi^	11.53 ± 0.51^fghi^	1.63± 0.06^gh^
150 µM Si+Ti +250 µM As	3.22± 0.24^bcd^	5.03± 0.26^cd^	8.25 ± 0.37^cd^	1.08 ± 0.06^bcd^
150 µM Zn+Si	7.11± 0.24^op^	9.05± 0.29^nop^	16.16 ± 0.51^opq^	2.97 ± 0.07^pq^
150 µM Zn+Si + 150 µM As	5.38± 0.30^ijk^	7.01± 0.32^ij^	12.39 ± 0.53^hijk^	1.89 ± 0.05^ij^
150 µM Zn+Si + 250 µM As	3.87± 0.28^def^	5.55± 0.29^def^	9.42 ± 0.57^de^	1.21 ± 0.06^cde^
150 µM Zn+Ti	6.96± 0.34^nop^	8.88± 0.34^no^	15.84 ± 0.62^opq^	2.86 ± 0.06^op^
150 µM Zn+Ti + 150 µM As	5.17± 0.27^ij^	6.78± 0.21^hi^	11.95 ± 0.47^ghij^	1.78 ± 0.08^hi^
150 µM Zn+Ti + 250 µM As	3.55± 0.30^cde^	5.23± 0.28^de^	8.03 ± 1.81^bcd^	1.16 ± 0.04^cd^
150 µM Si+Ti+Zn	7.65± 0.26^p^	9.78± 0.28^p^	17.43 ± 0.53^q^	3.12 ± 0.21^q^
150 µM Si+Ti+Zn + 150 µM As	7.24± 0.25^op^	9.35± 0.32^op^	16.59 ± 0.58^pq^	3.05 ± 0.08^pq^
150 µM Si+Ti+Zn + 250 µM As	6.75± 0.26^mno^	8.66± 0.32^mno^	15.41 ± 0.53^nop^	2.71 ± 0.06^no^

In this table, the letters a, b, c, etc. indicated significant differences following the application of NPs with 150 and 250 µM As based on Tukey′s test (p< 0.05).

### 3.8 Nanoparticles increase shoot and root dry weight as well as plant height under As toxicity

**Figure 6:**
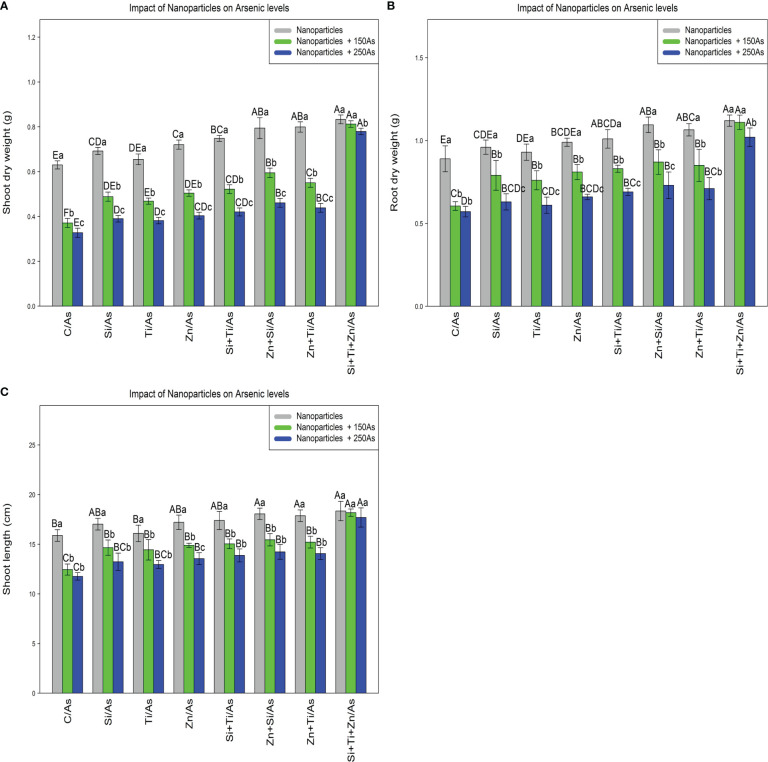
The impact of NPs alone and in combination on shoot dry weight **(A)**, root dry weight **(B)**, and shoot length **(C)** in one-year-old *Pleioblastus pygmaeus* exposed to 150 and 250 µM As (four replicates). In this figure, the letters a, b, c, *etc.* indicated significant differences following the application of NPs with 150 and 250 µM As based on Tukey′s test (p< 0.05).

The results revealed a statistically significant difference among different types of NPs alone and in combination with As concentrations (150 and 250 µM)(p<0.001). According to the results, the greatest increase in the plant growth and biomass was associated with a combination of three types of NPs at 150 and 250 µM As, with 32%, 29%, and 24% increase in shoot dry weight, 25%, 24%, and 14% increase in root dry weight, and 15%, 14%, and 11% increase in shoot length, respectively, while the treatments with 150 and 250 µM As resulted in the lowest amounts of dry weight of shoot (42% and 45%), dry weight of root (32% and 35%), and shoot length (21% and 26%) compared to the control treatment (**Figure 6**).

## 4 Discussion

Arsenic is recognized as a non-essential metalloid for plant growth. Nonetheless, As can accumulate at toxic levels in plant organs, which can subsequently contaminate the human food chain (Farooq et al., 2016; Zhang et al., 2021). Therefore, there is a great need for study in this field in order to develop novel techniques for reducing or removing arsenic’s environmental toxicity. Recent studies have demonstrated that nanomaterials are efficient sorbents for removing metal ions from plants and wastewater (Lu et al., 2016; Ethaib et al., 2022);. There is a physical interaction between NPs as sorbents and metal ions, therefore, NPs can inhibit metal ion mobility and translocation in plant organs and wastewater through absorbance (Tripathi and Ranjan, 2015). The NPs with high adsorption capacity can act as the binder of metal ions (Ethaib et al., 2022). NPs can enter the plant through root tissues or aerial parts (Ruttkay-Nedecky et al., 2017) and get accumulated into larger aggregates or micron-scale, which may significantly decrease the specific surface area of As and diminish its sorption capacity (Habuda-Stanić and Nujić, 2015). It has been reported that the cellular layers in roots including epiblema, endodermis, and exodermis are involved in the formation of apoplastic barriers in roots, providing a restrictive path for the uptake of heavy metals by plants. Meanwhile, NPs can contribute to the reduced metal uptake by plants *via* boosting the apoplastic defense barriers in the roots and increasing their impermeability (Aguirre-Becerra et al., 2022). Our results indicated that 150 µM of (TiO_2_ NPs, ZnO NPs, and SiO NPs) remarkably reduced the accumulation of As in the roots, stems, and leaves in the bamboo species. Therefore, the addition of NPs reduced As uptake by the bamboo roots through some mechanisms, such as adsorption and absorption of As ions as well as strengthening of the apoplastic defense barriers. This can result in a reduction of ion translocation to the aerial parts and decreased accumulation of As in the shoot and stem. This could also explain the decreasing bioaccumulation and translocation factors in this experiment. TF and BAF are two crucial indices for determining plant tolerance under toxicity (Radziemska, 2018; Usman et al., 2019). Hence, they can provide an assessment of the efficiency of employing NPs in terms of reducing As toxicity in the bamboo species. According to the findings of this study, with the addition of NPs, As concentration was diminished in the roots and shoots. The results of a study on garlic plants indicated that silicon NPs co-precipitated with metal ions, leading to the immobilization of metal ions and inhibition of the uptake of metal ions by the plants (Silva et al., 2017). Therefore, the co-precipitation of NPs with metal ions may be an effective pathway in minimizing the lethal accumulation of metals in plant tissues. On the other hand, our findings revealed that the combination of three NPs enhanced the NPs’ efficiency in removing heavy metals. Therefore, we suggested that, in addition to the absorbance mechanism, the number of NPs with a propensity for larger accumulation and aggregation in micron-scale reduces As sorption capacity and its effective surface area, thereby decreasing As toxicity in the bamboo plant organs. The reduction of accumulation and translocation of As in plants has been reported by ZnO NPs in rice (Yan et al., 2021), by SiO_2_ NPs in tomato (González-Moscoso et al., 2022), and by TiO_2_ NPs in rice (Wu et al., 2021). Glutathione, as a thiol compound, plays a substantial role in increasing plant tolerance to stressful conditions, which can influence multifarious biochemical processes and promote plant adaptation to stress (Vezza et al., 2019). When plants are exposed to the metal ions’ toxicity, GSH is reduced as an antioxidant agent and acts as a precursor of phytochelatins (PCs) within plant cells (Jung et al., 2019). PCs that use GSH as non-protein thiols (NPTs) for synthesis are able to produce chelates with arsenite (As(III)) *via* thiolate bonds (Souri et al., 2020). PCs synthase is the only self-regulatory enzymatic process that is triggered in plants exposed to toxic metals and metalloids (Memon et al., 2001). They contain a group of peptides that bind metal/metalloids in plants in response to the absorption of toxic metal ions (Ogawa et al., 2011). Our results indicated that, while the presence of As reduced the content of GSH and PCs in the bamboo plant, the addition of NPs increased the synthesis of PCs and GSH content. Therefore, we suggested that the addition of NPs can aid in the reduction of As toxicity by promoting the chelate formation of GSH and PCs with As (III). The PC-As (III) complexes are compartmentalized in the vacuoles, therefore that is one of the main As detoxification mechanisms in plants, together with As extrusion (Zanella et al., 2016). The role of PCs and GSH in increasing different plant species’ resistance to heavy metal stress has been established (Aborode et al., 2016; Souri et al., 2020).

On the other hand, AsA is one of the most significant natural antioxidants that can be oxidized to DHA. Consequently, these two molecules can form an efficient redox system that protects plant cells from ROS under stressful conditions (Yin et al., 2022). Additionally, AsA can boost plant photochemical activity through promoting photosynthesis (Olkhovych et al., 2016). The high concentration of AsA in the plant could be a positive indicator of the plant’s defense system capacity to respond to environmental stress. (Zelenchukova et al., 2015). In the present study, the addition of NPs increased the tolerance in the bamboo plant exposed to As toxicity, which we believe may be a result of AsA content as an antioxidant in the redox system. Under stress, plants produce proline and soluble sugars, which are two forms of osmolytes that can be involved in osmotic adjustment and detoxification of ROS (Mishra et al., 2016). Improving protein structure, preserving cellular osmolality, redox homeostasis, and most importantly, scavenging ROS in plants exposed to heavy metal toxicity are the results of an increase in sugars-protein complex and accumulation of proline (Ghorbani et al., 2018). Our results indicated that the combined application of NPs (ZnO,TiO_2_, and SiO_2_) remarkably increased the sugars-protein complex and proline content in the bamboo species under 150 µM and 250 µM As. Numerous studies have demonstrated that the application of NPs increases the sugars-protein complex and proline content (Elanchezhian et al., 2017; Tanveer et al., 2022), leading to the scavenging of ROS and the amelioration of oxidative stress in a variety of plant species.

The formation of ROS in plants by heavy metals has a detrimental impact on plant metabolism. The effects of ROS on the cellular metabolism are due to the stimulation of the Haber-Weiss reaction, which can induce lipid peroxidation of cell membrane, leading to the generation of hydroxyl radicals, and ultimately the disruption of membrane permeability and function (Mittler et al., 2012). ROS can also harm all the macromolecules in living species (Shields et al., 2021). In the present study, the results demonstrated that the high levels of As increased the ROS compounds (H_2_O_2_ and ${\text{O}}_{2}^{•-}$ ) and the lipid peroxidation indicator (MDA) in the bamboo plant, which is associated with oxidation in the bilayer membrane of lipids. Mousavi and Ahmad (Ahmad et al., 2020; Mousavi et al., 2020) showed similar As-induced effects on rice and beans, respectively. Since, MDA, as a lipid peroxidation indicator, is correlated with oxidative stress in a variety of plants exposed to stressful conditions, the presence of MDA demonstrates damage to the plant cell membrane (Morales and Munné-Bosch, 2019). Electrolyte leakage is an important index for determining plant stress tolerance, which indicates the extent to which intact plant cell membranes are damaged. (Senthilkumar et al., 2021). The MDA content and percentage of EL in this study suggested that the addition of NPs application reduced oxidative stress in the bamboo plants exposed to As toxicity, as demonstrated by a significant reduction in the MDA and EL content. Similar outcomes have been reported for *P. Vulgaris* plants exposed to cadmium stress (Koleva et al., 2022) and *Salvia splendens* exposed to various types of heavy metals (Faiz et al., 2021). Moreover, plants can minimize membrane lipid peroxidation and ROS accumulation *via* keeping their antioxidant enzymes at high levels (Hasanuzzaman et al., 2020). It has been reported that the direct binding of As to thiol groups can inhibit the stimulation of antioxidant activity, hence, intensifying oxidative stress in plants (Sharma, 2012). Our results demonstrated that NPs alone and in combination improved antioxidant activity, including SOD, CAT, POD, GR, and APX, leading to the reduced As toxicity in the bamboo species. In a study on grass pea, the up-regulation of SOD, CAT, and APX genes reduced oxidative stress caused by As (Talukdar, 2013). Enhancement of antioxidant activities in heavy metal-stressed plants by using NPs has been reported in bamboo plants treated with TiO_2_ NPs (Emamverdian et al., 2021b), and SiO_2_ NPs (Emamverdian et al., 2020), and in wheat (*Triticum aestivum*) treated with ZnO NPs (Hussain et al., 2018). When a plant is exposed to abiotic stress, the expression of two enzymes, Gly I and Gly II, can decrease MG levels (Singla-Pareek et al., 2008). This is accomplished in two steps *via* the regulatory role of the co-factor GSH. In the first step, which involves the formation of S-D-lactoylglutathione (SLG), Gly l catalyzes the non-enzymatic production of hemithioacetal from MG and GSH. In the next step, SLG catalyzes the subsequent release of D-lactate and regeneration of GSH, which is facilitated by Gly II (Yadav et al., 2005b; Hasanuzzaman et al., 2017). Hence, the glyoxalase system (Gly I and Gly II) is required for the conversion of MG to D-lactate and the recovery of GSH under stressful conditions (Hasanuzzaman et al., 2011a; Hasanuzzaman et al., 2011b). Our results indicated that the MG content decreased with increasing glyoxalase system when the As-exposed bamboo plants were treated with NPs, which demonstrated the efficiency of NPs in the stimulation of the glyoxalase system in the stressed bamboo plants. Some studies reported that MG detoxification through the glyoxalase system may reduce plant oxidative stress (Hasanuzzaman et al., 2011b; Hasanuzzaman and Fujita, 2013).

**Figure 7:**
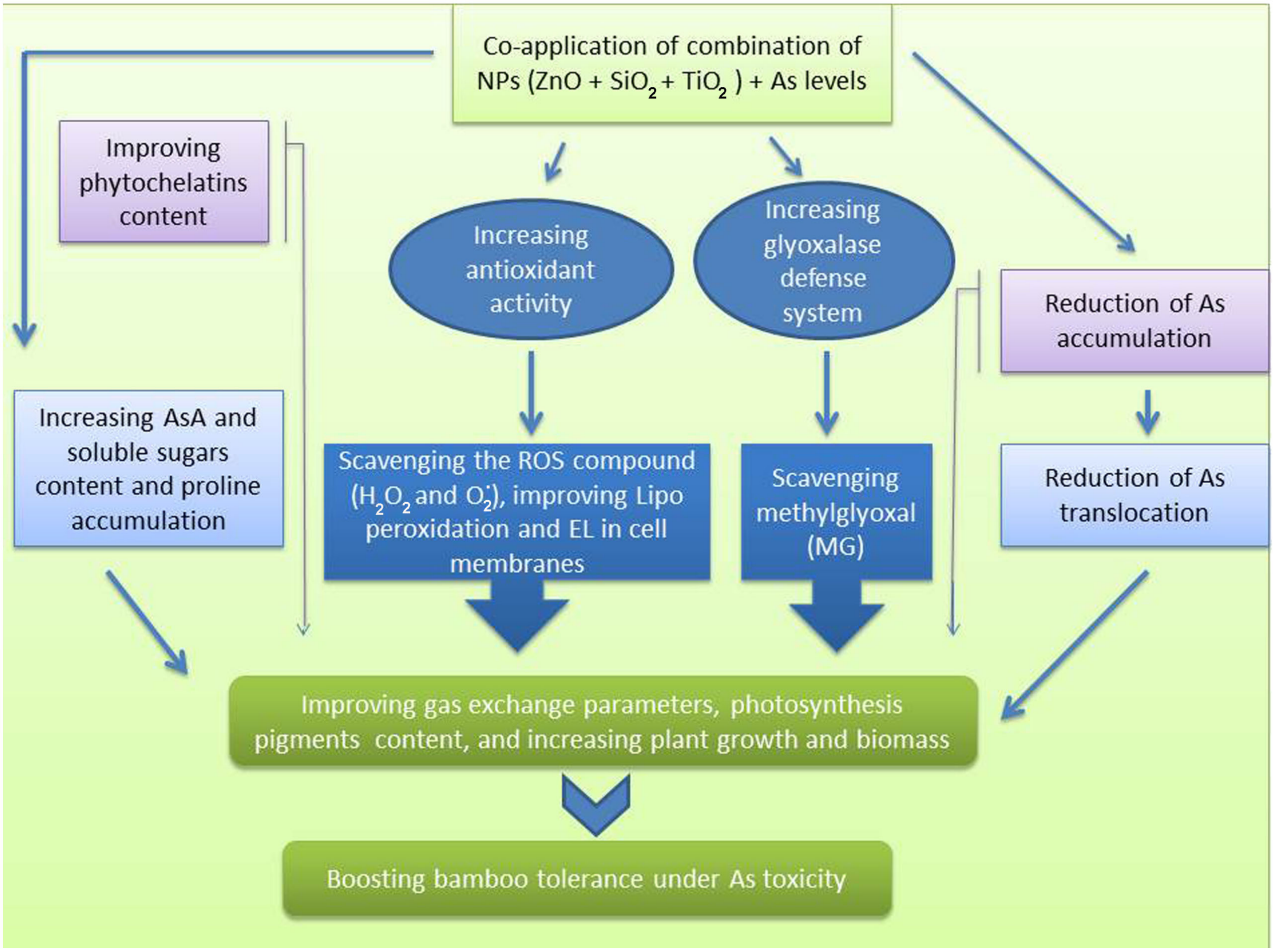
A mechanistic approach to demonstrate the actual mechanisms of SiO_2_ NPs, ZnO NPs, and TiO_2_NPs under Arsenic toxicity in bamboo.

Chlorophyll is one of the main chloroplast components that plays an essential role in the efficiency of photosynthesis in various plant species (Rizwan et al., 2017; Mandal and Dutta, 2020). In fact, they serve as an indicator for plant defense response to stressful conditions. Our results showed that the As levels lowered the content of photosynthetic pigments, gas exchange parameters, and chlorophyll fluorescence in the bamboo plants, which can be associated with a decrease in chlorophyll biosynthesis enzymes such as δ-ALAD activity and an increase in Chlase activity (Ghorbani et al., 2021; Salavati et al., 2021). In addition, As accumulation in leaves disrupts numerous physiological processes, including ATP and tetrapyrrole biosynthesis, and PSII photochemistry (Patel et al., 2018; Jung et al., 2019). On the other hand, NPs boosted the photosynthetic pigments, gas exchange parameters, and chlorophyll fluorescence in the present study. The ZnO NPs, SiO_2_ NPs and TiO_2_ NPs have been shown to increase photosynthetic pigments in various plants such as in *Leucaena leucocephala*, rice and wheat (Venkatachalam et al., 2017a; Venkatachalam et al., 2017b; Elshayb et al., 2021; Irshad et al., 2021). Increasing the chlorophyll content through the application of various NPs can increase the uptake and absorption of water by certain plants (Khan et al., 2020). For instance, silicon helps plants maintain a high water capacity under stressful conditions (Zhu and Gong, 2014). The application of NPs may result in enhanced photosynthesis efficiency in plants *via* the amelioration of oxidative stress (Adrees et al., 2020; Khalid et al., 2022). In light of these findings, we proposed that the application of NPs alone and in combination had a substantial role in ameliorating photosynthetic efficiency in the bamboo plant exposed to As toxicity, leading to improved plant growth and biomass indices as demonstrated by an increase in the photosynthetic pigments and the parameters related to gas exchange, and chlorophyll fluorescence. Numerous studies have demonstrated the detrimental effect of As on plant biomass and plant growth (Ahmad et al., 2020; Mousavi et al., 2020). In an experiment on rice, Mousavi et al. (2020) showed that there was a positive correlation between As levels and the generation of oxidative stress, leading to a reduction in nutrient absorption, and translocation of some nutrients, which ultimately diminished rice yield (Mousavi et al., 2020). This may be one of the primary explanations for the decrease in the bamboo plant growth and biomass caused by As in this study. Some evidence suggests that NPs mitigate the toxicity of heavy metals (Baby et al., 2020; Zhou et al., 2020). Additionally, trace elements can stimulate growth in heavy metal-stressed plants through a process, which is known as the NPs dilution effect on heavy metals (Rizwan et al., 2017; Rizwan et al., 2019b). Alternatively, the NPs can increase the nutrient supply (Gudkov et al., 2020). For instance, silicon enhanced protein content (Hussain et al., 2019) and ZnO NPs elevated zinc levels in wheat (Hussain et al., 2018). In the present study, we demonstrated that the combined form of all three NPs remarkably increased the bamboo plant biomass and growth indices under As toxicity. Similar results were obtained by ZnO NPs on *Leucaena leucocephala* (Venkatachalam et al., 2017a; Venkatachalam et al., 2017b) and by TiO_2_ NPs on soybean exposed to Cd toxicity (Singh and Lee, 2016). Therefore, we recommend the application of the combined NPs to promote plant growth in the presence of arsenic toxicity (**Figure 7**).

## 5 Conclusions

In the current study, NPs demonstrated a strong influence on boosting plant tolerance to As excess. The primary mechanism involved was the function of NPs as scavengers of ROS and MG together with the stimulation of antioxidants and the glyoxalase system, leading to a reduction in ROS compounds as well as a diminution of membrane lipid peroxidation and membrane damage, which ultimately protected the plant cells. On the other hand, As ion accumulation and translocation to the aerial parts were reduced by the NPs through the metal ions absorption and precipitation, hence contributing to the phytochelatins synthesis in the plant organs. As a result, the bamboo photosynthetic pigments levels increased, and the plant growth indices improved compared to the controls. The hypothesis of whether any type of the NPs (individually or in combination) had a greater decreasing effect on As toxicity in the bamboo species was tested in this paper for the first time. We concluded that 150 μM ZnO NP played the greatest role in alleviating As toxicity, and also the combinations of the three NPs, as demonstrated by Zn+Si+Ti> Zn+Si> Zn+Ti> Si+Ti> Zn >Si >Ti, were respectively the most effective at reducing the content of As ions in the bamboo plant. Hence, these NPs can be utilized to reduce or eliminate toxins from the environment and aid in increasing the bamboo plant resistance when grown in the As-contaminated areas. Future studies can concentrate on removing heavy metal/toxic elements from the various plants in the highly contaminated areas by employing low-cost NPs-based methods.

## Data availability statement

The raw data supporting the conclusions of this article will be made available by the authors, without undue reservation.

## Author contributions

Conceptualization, AE, YD, MH and JB. Statistical analysis, AE, YL. Writing original draft and revised preparation, AE, YD, JB, FM, MH, YL, and GL. Investigation, AE, GL. Supervision, AE, YD, GL. Project Administration, AE, YD, MH. Funding Acquisition, YD, GL. English editing, JB and FM. All authors contributed to the article and approved the submitted version.
