# Supplementary material for: Insight into the biochemical and physiological mechanisms of nanoparticles-induced arsenic tolerance in bamboo
Source: Front Plant Sci. 2023 Mar 31;14:1121886. doi: 10.3389/fpls.2023.1121886 (PMC10102603; doi:10.3389/fpls.2023.1121886)
Supplement: Supplementary file 2 [file DataSheet_2.docx]

Supplementary Material

**Insight into the biochemical and physiological mechanisms of nanoparticles-induced arsenic tolerance in bamboo**

**Abolghassem Emamverdian, Yulong Ding, Mirza Hasanuzzaman, James Barker, Guohua Liu, Yang Li, Farzad Mokhberdoran**

*** Correspondence:** Corresponding Author:

[mhzsauag@yahoo.com](mailto:mhzsauag@yahoo.com) (M.H)

## Supplementary Figures


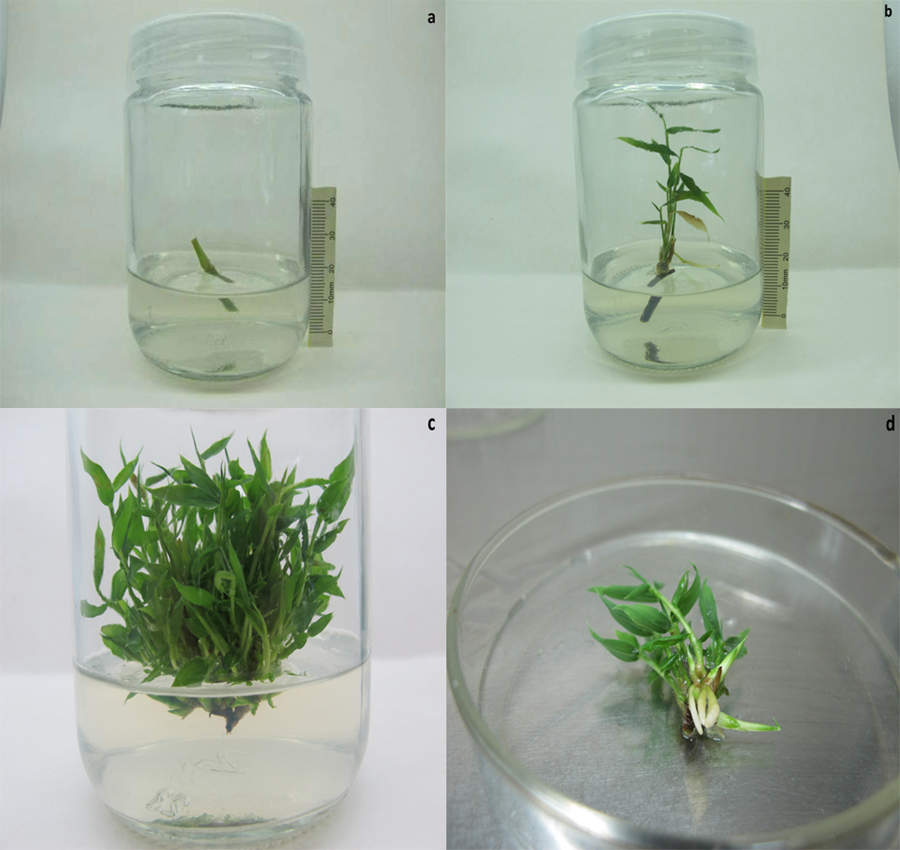


**Supplementary Figure 1.** Bamboo (*Pleioblastus pygmaeus*) growth *in vitro* culture a) 10 mm long nodal single clone 1-year-old bamboo species (*Pleioblastus pygmaeus*) b) bamboo treatments in tissue culture chamber 3 days after plantation c) bamboo treatments in tissue culture chamber three weeks after plantation d) bamboo treatments in Air Tech incubation hood before plantation in MS.

## Supplementary Tables

**Supplementary Table 1.** The experimental design.

| Treatments | Concentrations |
| --- | --- |
| Control | 0 |
| As | 150 µM As |
| As | 250 µM As |
| SiO_2_NPs | 150 µM SiO_2_NPs |
| SiO_2_NPs + As | 150 µM SiO_2_NPs +150 µM As |
| SiO_2_NPs + As | 150 µM SiO_2_NPs +250 µM As |
| TiO_2_NPs | 150 µM TiO_2_NPs |
| TiO_2_NPs +As | 150 µM TiO_2_NPs +150 µM As |
| TiO_2_NPs +As | 150 µM TiO_2_NPs +250 µM As |
| ZnO NPs | 150 µM ZnO NPs |
| ZnO NPs +As | 150 µM ZnO NPs +150 µM As |
| ZnO NPs +As | 150 µM ZnO NPs +250 µM As |
| SiO_2_NPs+TiO_2_NPs | 150 µM (SiO_2_NPs+TiO_2_NPs) |
| SiO_2_NPs+TiO_2_NPs | 150 µM (SiO_2_NPs+TiO_2_NPs)+150 µM As |
| SiO_2_NPs+TiO_2_NPs | 150 µM (SiO_2_NPs+TiO_2_NPs)+250 µM As |
| ZnONPs+SiO_2_NPs | 150 µM (ZnONPs+SiO_2_NPs) |
| ZnONPs+SiO_2_NPs + As | 150 µM (ZnONPs+SiO_2_NPs) +150 µM As |
| ZnONPs+SiO_2_NPs + As | 150 µM (ZnONPs+SiO_2_NPs) +250 µM As |
| ZnONPs+TiO_2_NPs | 150 µM (ZnONPs+TiO_2_NPs) |
| ZnONPs+TiO_2_NPs + As | 150 µM (ZnONPs+TiO_2_NPs) +150 µM As |
| ZnONPs+TiO_2_NPs + As | 150 µM (ZnONPs+TiO_2_NPs) +250 µM As |
| SiO_2_NPs+TiO_2_NPs+ ZnONPs | 150 µM (SiO_2_NPs+TiO_2_NPs+ ZnONPs) |
| SiO_2_NPs + TiO_2_NPs + ZnONPs +As | 150 µM (SiO_2_NPs+TiO_2_NPs+ ZnONPs) +150 µM As |
| SiO_2_NPs + TiO_2_NPs + ZnONPs +As | 150 µM (SiO_2_NPs+TiO_2_NPs+ ZnONPs) +250 µM As |
